# Comparison of Efficacy between Acupuncture Therapies in Improving Sacroiliac Joint Malposition: A Systematic Review and Meta-Analysis

**DOI:** 10.1155/2022/9485056

**Published:** 2022-01-11

**Authors:** Liguo Liu, Litao Pan, Minne Tian, Xiuhua Chen, Liming Lu, Guofeng Xu, Jian Sun, Yu Kui

**Affiliations:** ^1^The Second Clinical College of Guangzhou University of Chinese Medicine, Guangzhou, China; ^2^The First Affiliated Hospital of Shenzhen University, Shenzhen, China; ^3^The First Clinical College of Guangzhou University of Chinese Medicine, Guangzhou, China; ^4^Clinical Research Center, South China Research Center for Acupuncture and Moxibustion, Medical College of Acu-Moxi and Rehabilitation, Guangzhou University of Chinese Medicine, Guangzhou, China

## Abstract

**Aim:**

To provide available quantitative evidence of efficacy and safety of acupuncture treatments for improving sacroiliac joint malposition.

**Methods:**

Databases such as the China National Knowledge Infrastructure (CNKI), China Science and Technology Journal Database (CQVIP), Wanfang Database (Wanfang), China Biology Medicine disc (CBMdisc), PubMed, Web of Science, EMBASE, and Cochrane Library were searched by computer to collect the reports on acupuncture treatment of sacroiliac joint malposition from the database creation to July 20, 2021. The selection of included studies, data extraction and coding, and bias risk assessment were conducted independently by two reviewers. RevMan5.4 software was used for meta-analysis, and the results were expressed as mean difference (MD) or standardized mean difference (SMD), with a confidence interval (CI) of 95%.

**Results:**

A total of 10 randomized controlled clinical trials (RCTs) with 1019 participants were included. Their overall quality of methodology was not high, and there may be publication bias. Meta-analysis showed that the total effective rate of the treatment group was higher than that of the control group (OR = 2.74, 95% CI 2.00 to 3.74, *P* < 0.00001). The treatment group was better than the control group in improving VAS score (WMD = −1.56, 95% CI -2.18 to -0.94, *P* < 0.00001). The ODI score of the treatment group was lower than that of the control group (WMD = −6.04, 95% CI -7.05 to -5.02, *P* < 0.00001). With the improvement of the JOA score, the difference of iliac transverse diameter of sacroiliac joint dislocation and the index of sacroiliac joint malposition in the treatment group were better than those in the control group (*P* < 0.05). There was no significant heterogeneity among the studies.

**Conclusion:**

Acupuncture may have therapeutic advantages in improving sacroiliac joint malposition. Acupuncture and acupotomy provide a safe way to improve the related clinical symptoms and functional disorders in activity of sacroiliac joint dislocation. However, due to the low quality of the included literature, this conclusion still needs to be further verified by more high-quality and large-sample RCTs.

## 1. Introduction

Sacroiliac joint dysfunction is an underestimated cause of low back and hip pain, which is considered to affect 15% to 30% of patients with chronic pain [1]. With the in-depth study of the sacroiliac joint, its role as a pain generator in patients with spinal diseases has been better explained [2]. The occurrence of sacroiliac joint pain is usually caused by the interruption of the anatomical structures in the joint. A study shows that for about 75% sacroiliac joint pain in patients with chronic pain, specific causes can be found, with sacroiliac joint malposition being one of the important factors [3]. Sacroiliac joint malposition is more common in pregnant women, and the incidence rate is as high as 17%-25% [4], which may be caused by pelvic dilatation and increased exercise during pregnancy [5]. The clinical manifestations include low back pain, pelvic girdle pain, and pelvic floor dysfunction. Sacroiliac joint pain is more difficult to treat, and improper treatment can easily aggravate the already existing symptoms. Regarding the clinical symptoms and movement disorders caused by sacroiliac joint malposition, the treatment generally starts from nonsurgical intervention, including physical therapy, functional exercise, tuina, manual reduction, acupuncture, and moxibustion therapy. Severe cases require surgical treatment such as radiofrequency denervation or minimally invasive sacroiliac arthrodesis [2], but surgical treatment is not widely accepted by the public because of its sequelae and high cost. At present, the aim of treatment for sacroiliac joint malposition is to relax the soft tissues and muscles around the sacroiliac joint and eliminate the compression of nerves and muscles, which can help to reset the dislocated joint to the normal anatomical space, relieve pain symptoms, and restore joint function [6].

Acupuncture, which originated in China, is a green therapy based on the concept of Yin-yang and Qi circulation. Acupuncture mainly exerts its analgesic effects by stimulating the nervous system, producing local effects on local retrograde axonal reflexes, and releasing opioid peptide and 5-hydroxytryptamine [7]. At the same time, needles entering the human body causes slight trauma to the local area. This can promote the increase of local blood flow signal, improve local metabolism, and promote the recovery of soft tissue function [8]. As a traditional Chinese medicine (TCM) treatment, acupuncture has satisfactory clinical efficacy with simple manipulation and less adverse reactions, which has been widely used to treat various kinds of pain, including sacroiliac joint pain.

Many RCTs have been conducted to evaluate the effectiveness of acupuncture in improving sacroiliac joint dysfunction, but high-quality evidence to persuade more doctors to adopt this treatment is still lacking. Meta-analysis, the highest level of evidence, can provide a possible ranking for clinical treatment of sacroiliac joint malposition. As far as we know, no systematic analysis has been carried out on clinical trials of acupuncture in the treatment of sacroiliac joint malposition. Therefore, from the perspective of evidence-based medicine (EBM), this study adopted the meta-analysis method to explore the clinical efficacy and safety of acupuncture in improving sacroiliac joint malposition, to provide a more objective EBM basis for clinical treatment.

## 2. Materials and Methods

### 2.1. Search Strategy

Databases such as the China National Knowledge Infrastructure (CNKI), China Science and Technology Journal Database (CQVIP), Wanfang Database (Wanfang), China Biology Medicine disc (CBMdisc), PubMed, Web of Science, EMBASE, and Cochrane Library were searched by computer to collect the literature on acupuncture treatment of sacroiliac joint dislocation from the creation of the database to July 20, 2021, with the keyword retrieval method mainly adopted. The following search terms were used in Chinese and English: (Acupuncture OR Electroacupuncture OR Acupotomy OR Needle) AND (Sacroiliac joint malposition OR Sacroiliac arthritis OR Pelvic malposition OR Pelvic rotation OR Pelvic girdle pain) AND (Randomly OR Randomized controlled trial). The retrieval strategy was adjusted according to the situation of each database, and the detected documents were managed by Note-Express software. The details of the search strategy can be found in Supplementary S1.

### 2.2. Inclusion and Exclusion Criteria

The included studies met the following criteria: age above 18, regardless of race. The diagnosis of sacroiliac joint malposition must meet at least one of the following internationally or domestically authorized diagnostic criteria: the relevant diagnostic criteria of “sacroiliac joint malposition” in the guidelines for the diagnosis and treatment of common diseases of TCM chiropractic surgery [9] formulated by the Chinese Society of Traditional Chinese Medicine in 2012; the relevant diagnostic criteria of “sacroiliac joint injury” in the diagnostic efficacy standard of TCM [10] formulated by the State Administration of Traditional Chinese Medicine; the relevant diagnostic criteria of “sacroiliac joint malposition” in the European diagnosis and treatment guidelines for pelvic girdle pain [11]; and relevant diagnostic criteria for “sacroiliac joint malposition” in Diagnosis and Manual Treatment of Sacroiliac Joint Malposition [12].

The excluded studies met one of the following: (1) literature types: clinical trials without randomized grouping; documents with incomplete data or infeasibility in data extraction; if the conference paper and periodical paper were repeated, or the contents of Chinese paper and English paper were repeated, the one with higher quality was selected; (2) subjects: no explicit diagnostic criteria or using self-made criteria; (3) intervention: main intervention of the control group was a nonacupuncture one with acupuncture also used (including acupuncture, auricular point, and acupoint application); the experimental group contained interventions other than acupuncture therapy, but not for excluding the placebo effect of the control group; treatment course difference between the two groups, for example, the study with the treatment course of two weeks in the treatment group and four weeks in the control group should be excluded; (4) outcome indicators: the outcome indicators were inconsistent with the theme of this study.

### 2.3. Data Extraction and Quality Assessment

Two researchers collected and collated the literature independently and managed the obtained literature with Note-Express software. Firstly, the duplicated papers were eliminated. Then, by reading the title and abstract, papers that did not meet the inclusion criteria were eliminated. Finally, by carefully reading the full text, the remaining papers in accordance with the inclusion criteria were selected. Two researchers independently extracted data from the included literature, including the author's name, year of publication, sample size, intervention measures, treatment course, diagnostic criteria, outcome indicators, follow-up or not, existence of dropped participants or not, and adverse reactions. To ensure the integrity of the literature, contact was made with the authors in case of incomplete data. After the above process was completed, the results were cross-checked. In case of disagreement, the third researcher was consulted to reach an agreement. The whole process was documented with the PRISMA flowchart.

### 2.4. Outcome Indicators

The primary outcome indicator was the total effective rate. According to the improvement degree of clinical symptoms and functional activities, the clinical efficacy was divided into curative, markedly effective, effective, and ineffective. The total effective rate is calculated as the sum total of the number of the cured, the markedly effective, and the improved divided by the total number of participants.

Secondary outcome indicators need to include at least one of the following important outcome measures: Visual Analog Scale (VAS), Japanese Orthopaedic Association (JOA) score, Oswestry Disability Index (ODI), or any clearly defined objective research-specific criteria for distinguishing responders from nonresponders.

### 2.5. Bias Risk Assessment

The bias risk assessment tool of RCTs in the Cochrane systematic evaluation manual was used to evaluate the quality of the included literature. Two researchers independently evaluated “low risk,” “unclear risk,” and “high risk” for the following items: random allocation method, distribution concealment, blinding used by subjects, blinding used to evaluate the results, complete result data, whether there were reports of selective outcomes, and other bias sources. If there was disagreement in the evaluation process, the third researcher was consulted to reach an agreement.

### 2.6. Data Synthesis and Analysis

Review Manager 5.4 was used for meta-analysis. The heterogeneity of each study was tested. The data were assessed by the fixed-effect model when the statistical heterogeneity of the included studies was not significant (*I*^2^ < 50% and *P* > 0.05). Otherwise, the data was assessed by the random-effect model (50% ≤ *I*^2^ ≤ 75% and *P* < 0.05). Moreover, the source of heterogeneity was identified by sensitivity analysis or subgroup analysis. If there was obvious heterogeneity among the studies (*I*^2^ > 75% and *P* < 0.1), only descriptive analysis was performed. When the analysis index was a binary variable, the odds ratio (OR) was used as the effect scale index. When the number of studies included in the group was more than 10, the funnel chart was used to analyze the potential publication bias. When the analysis index was a continuous variable, the mean difference (MD) was used as the effect scale index to analyze the results of each combined effect and its 95% confidence interval (CI). When the number of studies included in the group was more than 10, the funnel chart was used to analyze the potential publication bias.

## 3. Results

### 3.1. Search Results

**Figure 1:**
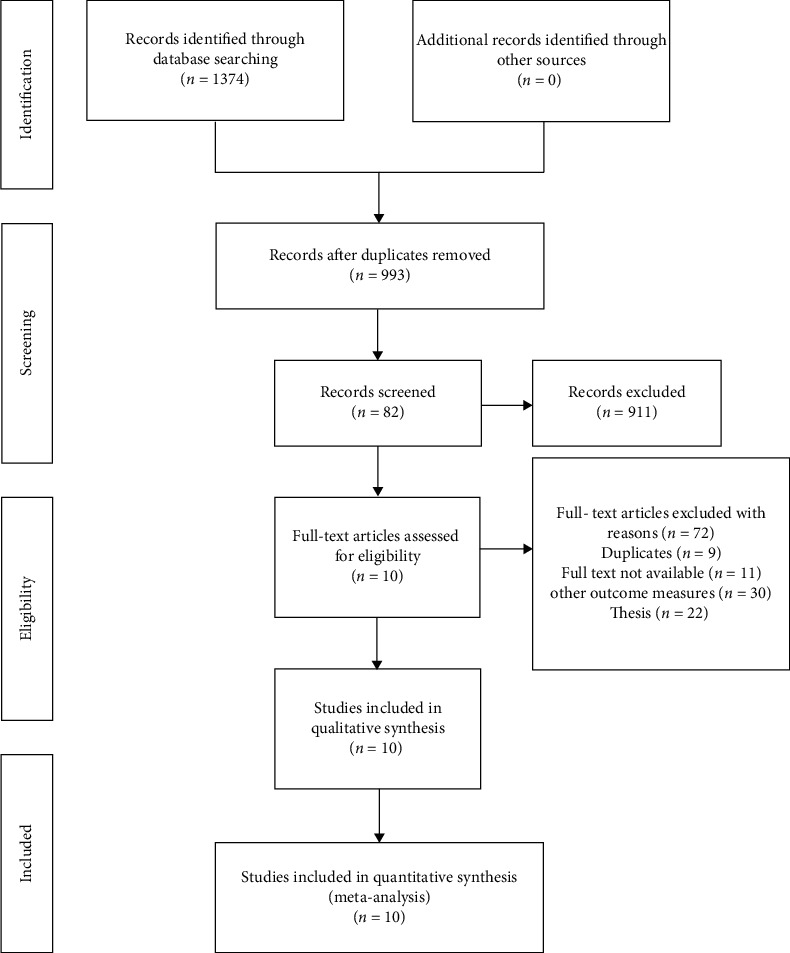
The PRISMA flow diagram.

A total of 1374 related studies were included in this systematic review, including 1192 from Chinese databases and 182 from foreign databases. After duplicate checking, 381 papers were eliminated. After reading their titles and abstracts, 911 unqualified ones were excluded. After searching and reading the full texts, 10 papers [13–22] were finally included with the total sample of 1019 participants. The process and results of literature screening are shown in Figure 1.

### 3.2. Study Characteristics of Included Studies

The included 10 papers [13–22] were RCTs including 1019 participants with 505 in the treatment group and 514 in the control group. Among them, for intervention of the treatment group, three studies [13, 17, 19] only used acupuncture, one study [16] used acupuncture combined with standard treatment, one study [22] used acupotomy, four studies [15, 18, 20, 21] used acupotomy combined with reduction manipulation, and one study [14] used acupotomy combined with reduction manipulation and muscle strength training. For the control groups, the interventions were mostly reduction manipulation. Among the outcome indicators, 10 studies [13–22] reported a total effective rate, five studies [13–16, 19] reported a VAS score, three studies [13, 14, 19] reported an ODI score, one study [15] reported a JOA score, one study [14] reported a transverse diameter of the iliac bone, and one study [17] reported a sacroiliac joint malposition index. The basic characteristics of the included studies are shown in Table 1.

### 3.3. Bias Risk Assessment

**Figure 2:**
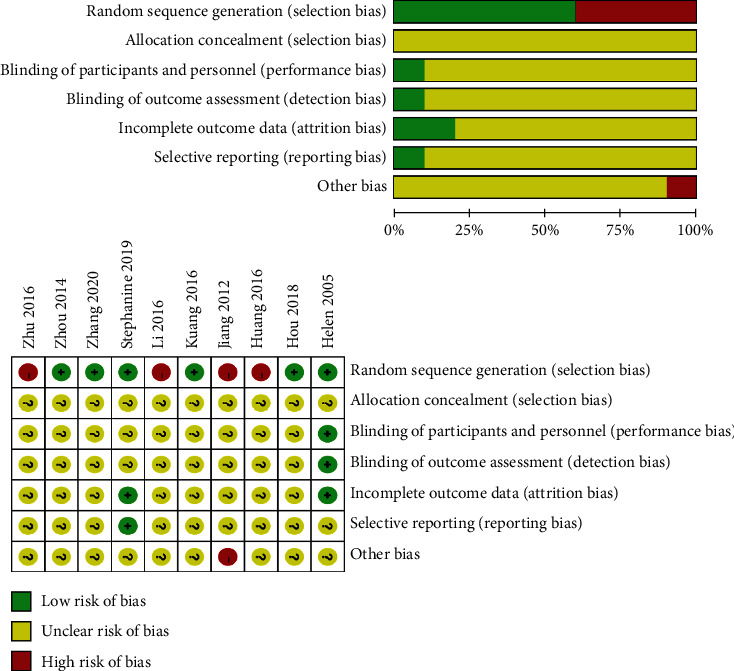
Risk of bias of all included studies.

The quality of the 10 papers included was evaluated by the bias risk assessment criteria provided by the Cochrane Collaboration Network. (1) *Random allocation method*: all 10 included studies [13–22] were RCTs, of which four studies [13–15, 17] used the random number table method, two studies [16, 19] used computer random number generators, and four studies [18, 20–22] did not describe a specific approach. (2) *Allocation concealment*: no allocation concealment schemes were mentioned in all the studies. (3) *Blinding*: except for one study [16], which clearly stated that blinding was not implemented, other studies did not mention whether blinding was established. (4) *Complete outcome data*: one study [16] mentioned the loss of follow-up and adopted intention analysis, while the numbers of samples included in the rest studies were consistent with the initial sample sizes, and the outcome data was complete. (5) *Selective outcome report*: data was insufficient to indicate whether there was selective bias. (6) *Other bias sources*: one study [20] did not describe the baseline data in detail, and the baselines of other studies were consistent. RevMan software was used to draw the bias risk assessment table (Figure 2).

### 3.4. Meta-Analysis

#### 3.4.1. Total Effective Rate

**Figure 3:**
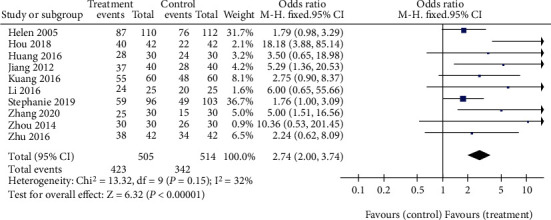
Forest plot for total efficacy rate.

**Figure 4:**
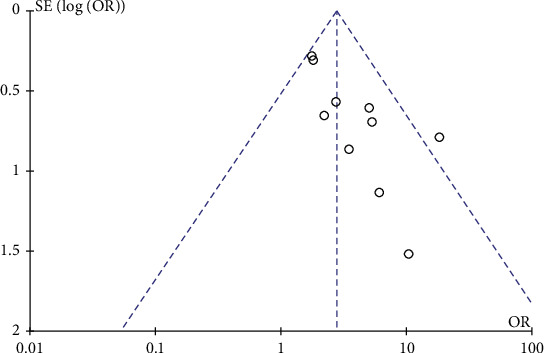
Funnel plot for publication bias for total efficacy rate.

A total of 10 studies [13–22] reported curative effect indicators such as curative, markedly effective, effective, and ineffective and set the sum of them as the total effective rate. The total effective rates of all treatment groups and control groups were tested for heterogeneity. The homogeneity among the studies was good (*P* = 0.15, *I*^2^ = 32%), so the fixed-effect model was used for analysis. Since the included data were binary variables, the M-H method was selected, and odds ratio (OR) and 95% confidence interval (CI) were used for calculation, as shown in Figure 3. Meta-analysis showed that the combined effect dose OR = 2.74 (95% CI 2.00 to 3.74), and the forest diagram showed that the effect test value *Z* = 6.32, *P* < 0.00001, indicating that acupuncture was better than nonacupuncture in improving sacroiliac joint malposition. The funnel chart of the total effective rate of the 10 studies showed that although all studies fell within the 95% CI, the lower left corner was slightly missing, indicating that there may be publication bias in the included studies (Figure 4).

**Figure 5:**
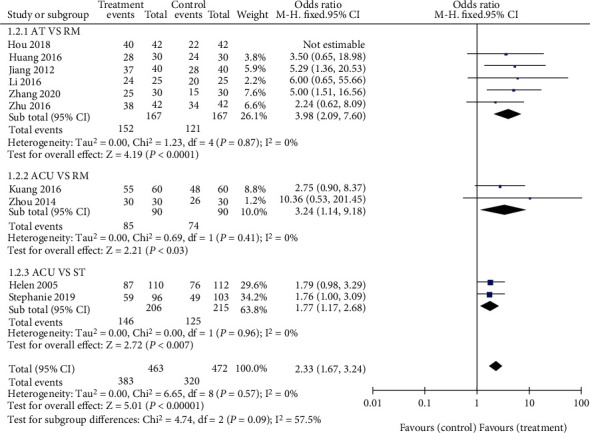
Forest plot for total efficacy rate subgroup analysis.

Subgroup analysis was conducted according to different intervention measures of the treatment group. It was found that in six studies [14, 15, 18, 20–22], the total effective rate of acupotomy combined with reduction manipulation was higher than that of reduction manipulation (167 participants in the treatment group and 167 in the control group). Two studies [13, 17] reported that the total effective rate of acupuncture was better than that of reduction manipulation (85 participants in the treatment group and 74 in the control group), and two studies [16, 19] reported that the total effective rate of acupuncture was higher than that of standard treatment or stability training (146 participants in treatment group and 125 in control group), with statistical significance (*P* < 0.05). It showed that acupuncture and acupotomy therapy may have more therapeutic advantages than other methods in the treatment of sacroiliac joint malposition (Figure 5).

#### 3.4.2. VAS Score

**Figure 6:**
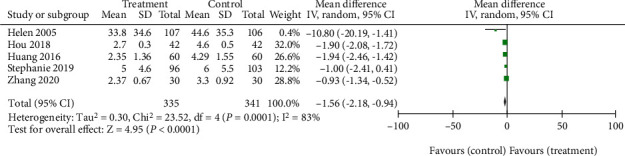
Forest plot for VAS score.

Five studies [14–16, 18, 19] compared the changes of the VAS score of acupuncture and reduction manipulation in the treatment of sacroiliac joint malposition. Because of different scoring methods, the total scores of VAS were different. Thus, they were divided into two subgroups for analysis. Among them, four studies [14, 15, 18, 19] adopted the 10-point scale, and the heterogeneity test revealed *P* = 0.0002, *I*^2^ = 85%, indicating that there was heterogeneity among the studies. Therefore, the random-effect model was adopted. It was found that for the total combined effect size, WMD = −1.53, (95% CI -2.11 to -0.94), *Z* = 5.11 with *P* < 0.00001, indicating statistical significance. The reasons for heterogeneity may be related to the low quality of the included studies or the inconsistency of interventions between groups. In addition, one study [16] adopted the 100-point system. The total combined effect size of the two subgroups showed WMD = −1.56 (95% CI - 2.18 to - 0.94), *P* < 0.00001, indicating that the acupuncture-based treatment group was better than the reduction-manipulation-based control group in improving the VAS score (Figure 6).

#### 3.4.3. ODI Score

**Figure 7:**
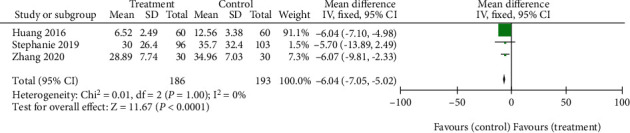
Forest plot for ODI score.

Three studies [14, 18, 19] compared the ODI score changes of the sacroiliac joint malposition between the treatment group and the control group. Since the included studies were homogeneous(*P* = 1.00, *I*^2^ = 0%), the combined effect size was calculated by a fixed-effect model. The results showed that the ODI score of acupotomy and acupuncture in the treatment of sacroiliac joint malposition was better than that of reduction manipulation and standard treatment (WMD = −6.04, 95% CI -7.05 to -5.02, *P* < 0.00001) (Figure 7).

#### 3.4.4. Other Outcome Indicators

One study [14] used the transverse diameter difference of ilium under X-ray as an evaluation index, and the improvement of the treatment group was better than that of the drug treatment group (*P* < 0.05). Another study [17] adopted the sacroiliac joint malposition index as the evaluation index and found that the improvement of the sacroiliac joint malposition in the acupuncture group was better than that in the manipulation group with statistical significance in the results (*P* < 0.05).

### 3.5. Adverse Events

Of the 10 RCTs, eight studies did not mention adverse events, and only two studies [13, 16] recorded adverse events caused by interventions. One study [16] mentioned that no adverse reactions occurred in either the treatment group or the control group. Another study [13] recorded that during the trial, three participants in the treatment group developed mild subcutaneous hematoma after receiving acupuncture.

## 4. Discussion

This study was a meta-analysis of acupuncture RCTs in the treatment of sacroiliac joint malposition. After searching the relevant literature published at home and abroad, 10 RCTs with 1019 participants were included according to the inclusion criteria for meta-analysis. In this paper, the efficacy of various treatment methods on clinical symptoms and signs caused by sacroiliac joint malposition were compared mainly from the aspects of total effective rate, VAS score, ODI score, JOA score, iliac transverse diameter difference, and sacroiliac joint malposition index, and several conclusions were made. Our results suggest that acupuncture and acupotomy therapy may be superior to manual reduction and functional exercise in improving the pain score and functional activity disorder of participants with sacroiliac joint malposition. This result was consistent with a previous study [23] which collected clinical trials of acupuncture and moxibustion in the treatment of low back pain. Through meta-analysis, it came to a conclusion that compared with drugs, tuina, and reduction manipulation, acupuncture alone has certain efficacy and advantages in improving pain symptoms and lumbar dysfunction in participants with lower back pain. According to that study, the mechanism may lie in acupuncture reduction in the excitability of nerve endings in lumbar tissue, promotion of muscle relaxation, and expansion of peripheral blood vessels. All together, they help to improve local microcirculation ischemia and hypoxia, eliminate inflammation and edema of local tissues, restore the normal biomechanical balance of the waist, reduce the symptoms of participants with low back pain, and improve the functional activities of the waist [24].

In terms of restoring the normal structure of the sacroiliac joint, the analysis of the two papers included in this study showed that the curative effect of acupuncture was better than that of manual reduction, but a different opinion was mentioned in a previous literature study which [25] evaluated the literature of the RCTs of manipulation in the treatment of sacroiliac joint malposition, with results that compared with acupuncture reduction; manipulation had a more significant and rapid effect in treating sacroiliac joint malposition, with significant immediate effects seen in some of the participants. Although the sacroiliac joint is an active joint of the pelvis, with its small range of motion, malposition is uncommon with the depressions and bulges that match with each other to stabilize the joint [26]. Only when a strong external force and long-term accumulated chronic strain exceed the normal bearing capacity will the corresponding relationship of the joint surface change slightly in anatomical position. This leads to the imbalance of the internal and external mechanical environment, resulting in injury, pain, and dysfunction of the local soft tissues [27]. As for treatment, although manual reduction can quickly bring the disordered joint back to its right position, its effect in treating the tension and contracture of local soft tissue is limited, resulting in a consequently high recurrence rate of the disease [28]. Acupuncture, on the one hand, can dredge the meridians, promote blood circulation, and relieve pain. On the other hand, it can loosen the local tendon nodes and adjust the channel sinews along the sacroiliac joint to restore the mechanical balance of the sacroiliac joint to correct the malposition of the sacroiliac joint [29].

Adverse event reports showed that only a few participants included in the study treated by acupuncture had mild subcutaneous hematoma. The occurrence of these events may be related to individual differences or acupuncturists' proficiency. In general, acupuncture is safe and effective in the treatment of sacroiliac joint malposition.

There are still some limitations in this study: (1) There are few related published studies (only 10 were included), and the included studies lack multicenter and large-sample RCT studies. (2) The methodological quality of the included literature is low. Most studies only mentioned the use of random methods without the operation of specific random methods or implementation of blinding and allocation hiding schemes, which greatly affects the demonstration strength of research results. (3) There are differences in the disease course, treatment time, specific acupoint selection, manipulation, and combined treatment methods among participants from different RCTs, which may well affect the results.

## 5. Conclusions

The results of this study showed that for the treatment of sacroiliac joint malposition, acupuncture had advantages over the control group in the total effective rate and the improvement of the VAS score, ODI score, JOA score, iliac transverse diameter difference, and sacroiliac joint malposition index, etc. This suggested that acupuncture including acupotomy might have curative effect advantages in improving sacroiliac joint malposition and could improve the related clinical symptoms and dysfunction of sacroiliac malposition with high safety. Yet, because of unsatisfactory quality of the included literature and problems of methodology and bias risk, this conclusion needs to be further verified by more high-quality and large-sample RCTs.

## Figures and Tables

**Table 1:** Basic characteristics of the included studies.

Study	Mean years (y)		Sample size		Intervention		Treatment duration	Outcomes	Follow-up	Side effects
	Treatment	Control	Treatment	Control	Treatment	Control				
Kuang 2016 [13]	38.4 ± 9.2	37.8 ± 8.7	60	60	ACU	RM	20 days	① ② ③	Not stated	3 patients reported minor hematoma in treatment group
Zhang 2020 [14]	38.4 ± 9.2	37.8 ± 8.7	30	30	AT, RM, MST	RM, MST	6 weeks	① ② ③ ⑤	Not stated	Not stated
Hou 2018 [15]	51.60 ± 4.01	51.53 ± 4.46	42	42	AT, RM	RM	4 weeks	① ② ④	Not stated	Not stated
Helen 2005 [16]	32.7 ± 8.7	33.2 ± 8.4	125	131	ACU, ST	SE, ST	6 weeks	① ②	After 1 week	Not happened
Zhou 2014 [17]	30.6 ± 4.0	30.0 ± 4.0	30	30	ACU	RM	10 days	① ⑥	Not stated	Not stated
Huang 2016 [18]	35.8 ± 8.7	34.4 ± 8.7	30	30	AT, RM	RM	15 days	①	Not stated	Not stated
Stephanie 2019 [19]	38.26 ± 2.37	39.27 ± 2.35	96	103	ACU	ST	4 weeks	① ② ③	After 1 week	Not stated
Jiang 2012 [20]	31.0 ± 5.2	30.7 ± 4.6	40	40	AT, RM	RM	3 weeks	①	Not stated	Not stated
Zhu 2018 [21]	—	—	42	42	AT, RM	RM	15 days	①	Not stated	Not stated
Li 2016 [22]	39.87 ± 3.35	40.15 ± 3.28	25	25	AT	RM	3 weeks	①	Not stated	Not stated

ACU: acupuncture; AT: acupotomy therapy; RM: reduction manipulation; MST: muscle strength training; ST: standard treatment; SE: stabilizing exercises; ①: total efficiency rate; ②: visual analog scale; ③: the Oswestry Disability Index; ④: Japanese Orthopaedic Association scores; ⑤: ilium transverse diameter differences; ⑥: sacroiliac joint movement index.

## Data Availability

The dataset can be accessed from the corresponding author upon reasonable request.

## Supplementary Materials

Supplementary Materials S1: searching strategies for all databases. S2: all 10 included studies. S3: PRISMA checklist.
