# Supplementary material for: Comparison of Efficacy between Acupuncture Therapies in Improving Sacroiliac Joint Malposition: A Systematic Review and Meta-Analysis
Source: Biomed Res Int. 2022 Jan 11;2022:9485056. doi: 10.1155/2022/9485056 (PMC8766180; doi:10.1155/2022/9485056)
Supplement: Supplementary Materials — S1: searching strategies for all databases. S2: all 10 included studies. S3: PRISMA checklist. [file 9485056.f1.zip › S1_Searching strategies for all databases.pdf]

TABLE 1: Searching strategy in PubMed

Search strategy
#1 “Acupuncture OR Electroacupuncture OR Acupotomy OR Needle” [Title/Abstract]
#2 “Sacroiliac joint malposition OR Sacroiliac arthritis OR Pelvic malposition OR Pelvic rotation OR Pelvic girdle pain OR Sacroiliitis” [Title/Abstract]
#3 “Randomly OR Randomized controlled trial ” [Title/Abstract]
#4 #1 AND #2 AND #3

TABLE 2: Searching strategy in EMBASE

Search strategy
#1 “Acupuncture OR Electroacupuncture OR Acupotomy OR Needle” [Title,Abstract,Author keywords]
#2 “Sacroiliac joint malposition OR Sacroiliac arthritis OR Pelvic malposition OR Pelvic rotation OR Pelvic girdle pain OR Sacroiliitis” [Title,Abstract,Author keywords]
#3 “Randomly OR Randomized controlled trial ” [Title,Abstract,Author keywords]
#4 #1 AND #2 AND #3

TABLE 3: Searching strategy in Web of Science

Search strategy
#1 “Acupuncture OR Electroacupuncture OR Acupotomy OR Needle” [Topic]
#2 “Sacroiliac joint malposition OR Sacroiliac arthritis OR Pelvic malposition OR Pelvic rotation OR Pelvic girdle pain OR Sacroiliitis” [Topic]
#3 “Randomly OR Randomized controlled trial ” [Topic]
#4 #1 AND #2 AND #3

TABLE 4: Searching strategy in Cochrane Library

Search strategy
#1 “Acupuncture OR Electroacupuncture OR Acupotomy OR Needle” [Title Abstract keywords]
#2 “Sacroiliac joint malposition OR Sacroiliac arthritis OR Pelvic malposition OR Pelvic rotation OR Pelvic girdle pain OR Sacroiliitis” [Title Abstract keywords]
#3 “Randomly OR Randomized controlled trial ” [Title Abstract keywords]
#4 #1 AND #2 AND #3

TABLE 5: Searching strategy in CNKI

Search strategy
#1 “Acupuncture OR Electroacupuncture OR Acupotomy OR Needle” [Title, Abstract, keywords]
#2 “Sacroiliac joint malposition OR Sacroiliac arthritis OR Pelvic malposition OR Pelvic rotation OR Pelvic girdle pain OR Sacroiliitis” [Title, Abstract, keywords]
#3 “Randomly OR Randomized controlled trial ” [Title, Abstract, keywords]
#4 #1 AND #2 AND #3

TABLE 6: Searching strategy in CBM

Search strategy
#1 “Acupuncture OR Electroacupuncture OR Acupotomy OR Needle” [Abstract]

#2 “Sacroiliac joint malposition OR Sacroiliac arthritis OR Pelvic malposition OR Pelvic rotation OR Pelvic girdle pain OR Sacroiliitis” [Abstract]
#3 “Randomly OR Randomized controlled trial ” [Abstract]
#4 #1 AND #2 AND #3

TABLE 7: Searching strategy in CQVIP

Search strategy
#1 “Acupuncture OR Electroacupuncture OR Acupotomy OR Needle” [Title, Abstract, keywords]
#2 “Sacroiliac joint malposition OR Sacroiliac arthritis OR Pelvic malposition OR Pelvic rotation OR Pelvic girdle pain OR Sacroiliitis” [Title, Abstract, keywords]
#3 “Randomly OR Randomized controlled trial ” [Title, Abstract, keywords]
#4 #1 AND #2 AND #3

TABLE 8: Searching strategy in Wanfang

Search strategy
#1 “Acupuncture OR Electroacupuncture OR Acupotomy OR Needle” [Topic]
#2 “Sacroiliac joint malposition OR Sacroiliac arthritis OR Pelvic malposition OR Pelvic rotation OR Pelvic girdle pain OR Sacroiliitis” [Topic]
#3 “Randomly OR Randomized controlled trial ” [Topic]
#4 #1 AND #2 AND #3
