# Supplementary material for: Comparison of Efficacy between Acupuncture Therapies in Improving Sacroiliac Joint Malposition: A Systematic Review and Meta-Analysis
Source: Biomed Res Int. 2022 Jan 11;2022:9485056. doi: 10.1155/2022/9485056 (PMC8766180; doi:10.1155/2022/9485056)
Supplement: Supplementary Materials — S1: searching strategies for all databases. S2: all 10 included studies. S3: PRISMA checklist. [file 9485056.f1.zip › S2_All included studies.pdf]

S2 All included studies

Kuang JY, Li YX, He YF, et al. Chinese Therapeutic Effect of Oblique Needling with Tuina in Relieving Sacroiliac Joint Injury [J]. Acupuncture Research, 2016, 41 (02): 169-174.

Zhang XG, Li XJ, Tang JH, et al. Clinical observation of needle knife combined with management and muscle strength training in the treatment of chronic sacroiliac joint dysfunction [J]. China Medical Herald, 2020, 17 (36): 163-166,196.

Hou XJ, Ma HM, Xiong W, et al. Clinical Study on the Treatment of Sacroiliac Joint Dislocation with Manipulation and Small Needle Knife [J]. Medical Innovation of China, 2018, 015 (032): 80-83.

Elden Helen, Ladfors L, Olsen MF, et al. Effects of acupuncture and stationing exercises as adjunct to standard treatment in pregnant women with pelvic girl pain: random single blind controlled trial [J]. BMJ, 2005, 330.

Zhou XK, Wei SS, Qin T, et al. Clinical Study on Treatment of Sacroiliac Joint Dislocation with Giant Needle at Baliao Point [J]. Journal of Youjiang Medical University for Nationalities, 2014, 36 (4).

Huang L, Yuan J, Hu SY, et al. Efficiency Observation of Massage and Acupotomy Treating Sacroiliac Injury [J]. Chinese Manipulation & Rehabitation Medicine, 2016, 007 (004): 34-35.

Nicolian S, Butel T, Gambotti L, et al. Cost-effectiveness of acupuncture versus standard care for pelvic and low back pain in pregnancy: A randomized controlled trial [J]. Plos One, 2019, 14 (4).

Jiang ZG. Observation on Therapeutic Effect of Needle Knife and Fine Adjustment Manipulation on Postpartum Sacroiliac Joint Malposition [J]. Clinical Journal of Traditional Chinese Medicine, 2012, 24 (010): 973-974.

Zhu MH. Treatment of Sacroiliac Joint Injury with Massage Manipulation and Needle Knife Clinical effect analysis [J]. Contemporary Medicine, 2018, 024 (005): 137-138.

Li L, Wang YW, Pang HB, et al. Observation on Therapeutic Effect of 50 Cases of Sacroiliac Joint Disturbance Treated by Ultra-micro Needle Knife [A]. Hunan University of Chinese Medicine Press, 2016: 2.
