# Supplementary material for: Comparison of Efficacy between Acupuncture Therapies in Improving Sacroiliac Joint Malposition: A Systematic Review and Meta-Analysis
Source: Biomed Res Int. 2022 Jan 11;2022:9485056. doi: 10.1155/2022/9485056 (PMC8766180; doi:10.1155/2022/9485056)
Supplement: Supplementary Materials — S1: searching strategies for all databases. S2: all 10 included studies. S3: PRISMA checklist. [file 9485056.f1.zip › S3_PRISMA checklist.pdf]

PRISMA 2009 Checklist

Section/topic	#	Checklist item	Reported on page #
**TITLE**			
Title	1	Identify the report as a systematic review, meta-analysis, or both.	Page 1 (**Title**)
**ABSTRACT**			
Structured summary	2	Provide a structured summary including, as applicable: background; objectives; data sources; study eligibility criteria, participants, and interventions; study appraisal and synthesis methods; results; limitations; conclusions and implications of key findings; systematic review registration number.	Page 1 (**Abstract**)
**INTRODUCTION**			
Rationale	3	Describe the rationale for the review in the context of what is already known.	Page 1, 2 (**Introduction**)
Objectives	4	Provide an explicit statement of questions being addressed with reference to participants, interventions, comparisons, outcomes, and study design (PICOS).	Page 1, 2 (**Introduction**)
**METHODS**			
Protocol and registration	5	Indicate if a review protocol exists, if and where it can be accessed (e.g., Web address), and, if available, provide registration information including registration number.	CRD42021265471
Eligibility criteria	6	Specify study characteristics (e.g., PICOS, length of follow-up) and report characteristics (e.g., years considered, language, publication status) used as criteria for eligibility, giving rationale.	Page 2 **(Inclusion and Exclusion Criteria)**
Information sources	7	Describe all information sources (e.g., databases with dates of coverage, contact with study authors to identify additional studies) in the search and date last searched.	Page 2 (**Search Strategy)**
Search	8	Present full electronic search strategy for at least one database, including any limits used, such that it could be repeated.	Page 10 (**Supplementary Materials**)
Study selection	9	State the process for selecting studies (i.e., screening, eligibility, included in systematic review, and, if applicable, included in the meta-analysis).	Page 2, 3 (**Data Extraction and Quality Assessment)**
Data collection process	10	Describe method of data extraction from reports (e.g., piloted forms, independently, in duplicate) and any processes for obtaining and confirming data from investigators.	Page 2, 3 (**Data Extraction and Quality Assessment)**
Data items	11	List and define all variables for which data were sought (e.g., PICOS, funding sources) and any assumptions and simplifications made.	Page 2, 3 (**Data Extraction and Quality**

			**Assessment)**
Risk of bias in individual studies	12	Describe methods used for assessing risk of bias of individual studies (including specification of whether this was done at the study or outcome level), and how this information is to be used in any data synthesis.	Page 3 (**Bias Risk Assessment)**
Summary measures	13	State the principal summary measures (e.g., risk ratio, difference in means).	Page 3 (**Data Synthesis and Analysis)**
Synthesis of results	14	Describe the methods of handling data and combining results of studies, if done, including measures of consistency (e.g., I^2) for each meta-analysis.	Page 3 (**Data Synthesis and Analysis)**

Section/topic	#	Checklist item	Reported on page #
Risk of bias across studies	15	Specify any assessment of risk of bias that may affect the cumulative evidence (e.g., publication bias, selective reporting within studies).	Page 3 (**Data Synthesis and Analysis)**
Additional analyses	16	Describe methods of additional analyses (e.g., sensitivity or subgroup analyses, meta-regression), if done, indicating which were pre-specified.	Page 3 (**Data Synthesis and Analysis)**
**RESULTS**			
Study selection	17	Give numbers of studies screened, assessed for eligibility, and included in the review, with reasons for exclusions at each stage, ideally with a flow diagram.	Page 3, 4 (**Search Results,** Fig. 1)
Study characteristics	18	For each study, present characteristics for which data were extracted (e.g., study size, PICOS, follow-up period) and provide the citations.	Page 3, 5 (**Study Characteristics of Included Studies**, Table 1)
Risk of bias within studies	19	Present data on risk of bias of each study and, if available, any outcome level assessment (see item 12).	Page 5(**Bias Risk Assessment,** Fig.2**)**
Results of individual studies	20	For all outcomes considered (benefits or harms), present, for each study: (a) simple summary data for each intervention group (b) effect estimates and confidence intervals, ideally with a forest plot.	Page 5, 7, 8, 9 (Table 1, Fig. 3, Fig. 5, Fig. 6, Fig.7)
Synthesis of results	21	Present results of each meta-analysis done, including confidence intervals and measures of consistency.	Page 6, 7, 8, 9 (**Meta-Analysis**, Fig. 3, Fig. 5, Fig.6, Fig.7)
Risk of bias across studies	22	Present results of any assessment of risk of bias across studies (see Item 15).	Page 7 (Fig. 4)
Additional analysis	23	Give results of additional analyses, if done (e.g., sensitivity or subgroup analyses, meta-regression [see Item	Page 8 (Fig. 5)

PRISMA 2009 Checklist

		16]).	
**DISCUSSION**			
Summary of evidence	24	Summarize the main findings including the strength of evidence for each main outcome; consider their relevance to key groups (e.g., healthcare providers, users, and policy makers).	Page 10, 11 (**Discussion**)
Limitations	25	Discuss limitations at study and outcome level (e.g., risk of bias), and at review-level (e.g., incomplete retrieval of identified research, reporting bias).	Page 10, 11 (**Discussion**)
Conclusions	26	Provide a general interpretation of the results in the context of other evidence, and implications for future research.	Page 10 (**Conclusions**)
**FUNDING**			
Funding	27	Describe sources of funding for the systematic review and other support (e.g., supply of data); role of funders for the systematic review.	Page 10 (**Acknowledgments)**

From: Moher D, Liberati A, Tetzlaff J, Altman DG, The PRISMA Group (2009). Preferred Reporting Items for Systematic Reviews and Meta-Analyses: The PRISMA Statement. PLoS Med 6(6): e1000097. doi:10.1371/journal.pmed1000097

For more information, visit: **www.prisma-statement.org**.
